# Recurrent Transient Hypoxemia in a Schizophrenic Patient Caused by Upper Airway Foreign Body Obstruction by Watermelon

**DOI:** 10.7759/cureus.48809

**Published:** 2023-11-14

**Authors:** Kaori Amari, Risa Hirata, Masaki Tago

**Affiliations:** 1 Department of Emergency Medicine, Saga-Ken Medical Centre Koseikan, Saga, JPN; 2 Department of General Medicine, Saga University Hospital, Saga, JPN

**Keywords:** diagnostic error, hypoxemia, airway obstruction, bias, schizophrenia

## Abstract

A woman in her 70s with schizophrenia experienced repeated episodes of limb tremors and hypoxemia. Even after admission, the same symptoms continued while in a supine position. However, her condition rapidly improved with bag valve mask ventilation. Although computed tomography suggested aspiration pneumonia, she had a strong cough reflex while performing bronchoscopy, and no residues were observed in the trachea. Following the bronchoscopy examination, the patient was prompted by a nurse and subsequently expelled a mass of watermelon from her oral cavity, which was identified as a watermelon eaten during breakfast on the day. She was diagnosed with aspiration pneumonia, and treatment with 2 g/day of cefotiam was initiated. The inflammatory response had improved, and she was transferred to another hospital for adjustment of psychiatric medications on the 10th day of admission.

A study indicated that 77% of emergency medical staff experienced misdiagnosis or delayed diagnosis of patients with mental illnesses. In the present case, various biases and system factors were found to be involved in the diagnostic error. It is crucial to recognize the potential for diagnostic errors in managing patients with schizophrenia given the various biases that may come into play. Furthermore, patients with schizophrenia are at high risk of upper airway foreign body obstruction because of dysphagia or drug-induced effects. When transient hypoxemia is observed, a prompt assessment of the visible intraoral region and, if necessary, evaluation of the entire upper airway through imaging studies should be considered.

## Introduction

Upper airway obstruction caused by a foreign body can potentially lead to fatal outcomes [1,2]. Foreign body-induced upper airway obstruction affects approximately 250 individuals annually in the United Kingdom, 5300 individuals in the United States, and 9200 individuals in Japan [2], making it the most common cause of accidental death in Japan [3-5]. The common culprits of airway foreign bodies include food items such as rice cakes, white rice, vegetables, meat, beans, bones, and teeth [6]. Particularly in patients with schizophrenia, dysphagia resulting from disease-related whole-food eating habits or drug-induced effects is considered a leading cause of upper airway obstruction [7]. Consequently, the mortality rate from acute asphyxia in schizophrenia patients is reported to be 100 times higher than that of the general population [7] and ranks as the third most common cause of sudden death, following myocardial infarction and pneumonia [8]. Furthermore, a study indicated that 77% of emergency medical staff (physicians/nurses) experienced misdiagnosis or delayed diagnosis of patients with mental illnesses [9]. Given the heightened vulnerability to diagnostic errors in patients with psychiatric disorders, including schizophrenia, it is essential to share actual cases experienced to prevent future diagnostic inaccuracies. We report here a case of schizophrenia with a delayed diagnosis of an upper airway foreign body that led to two episodes of transient hypoxemia.

## Case presentation

A woman in her 70s taking oral medication for schizophrenia was attending a daycare facility. At the facility, she went to the restroom after taking her medication following breakfast on day zero. Ten minutes later, facility staff found her in a state of semi-consciousness with her face resting on the toilet bowl. While the staff was in the process of trying to place the patient in a supine position, her level of consciousness improved. However, after being placed in a supine position, she exhibited one to two minutes of intermittent limb tremors five times. As the patient had a history of tremors due to an antipsychotic medication drug overdose during a dosage adjustment, a potential drug overdose was suspected, prompting the request for emergency medical services. Upon their arrival, the emergency responders and a physician conducted an examination but did not observe limb tremors, noted that unsmooth speech was present, and found no abnormalities in other vital signs. Therefore, the patient was suspected of being in a postictal state after a seizure and was transported to another hospital.

A head computed tomography (CT) scan was performed to assess intracranial lesions, but no abnormalities were observed. However, after the CT examination, while still on the scanning table, the patient developed left-sided gaze palsy and her respiratory rate rapidly decreased, leading to a decrease in oxygen saturation (SpO_2_) to lower than 50%. Immediate bag valve mask ventilation was administered, leading to an improvement in respiratory status. Prompt oxygen therapy was initiated, gradually improving her SpO_2_ to 97% (2 L nasal cannula). Owing to suspicion of a generalized seizure with accompanying hypoxemia, the patient was transferred to our hospital for further investigation.

**Figure 1:**
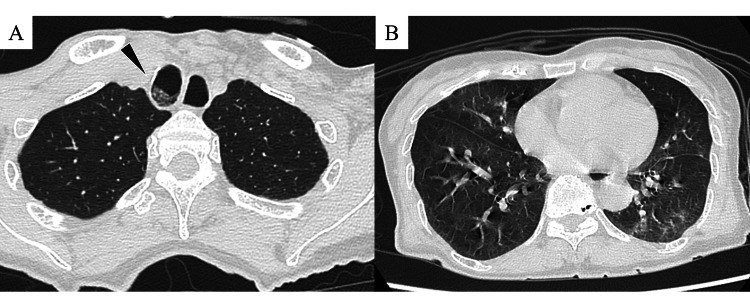
Chest computed tomography without contrast enhancement. Chest computed tomography without contrast enhancement showed intrabronchial fluid retention (arrowheads, A) and ground-glass opacities in the right middle lobe, left upper lobe, and lower lobe (B).

During the transfer from the previous hospital, the patient showed hyperactivity and excessive speech, restlessness, and repeated attempts to sit up, but no worsening of hypoxemia or seizure episodes was observed. On arrival at our hospital, she did not present with gaze deviation or limb paralysis but had preserved speech and continued with repeated attempts to sit up. Vital signs were as follows: Glasgow Coma Scale of E4V4M6, respiratory rate of 24 breaths per minute, SpO_2_ at 97% (2 L per minute with the nasal cannula), heart rate of 100 beats per minute, blood pressure of 143/75 mmHg, and body temperature of 37.4°C. Despite the patient’s restlessness, the accompanying facility staff informed the medical team that there was no notable change in her usual behavior. On chest auscultation, no crackles were present, and rhonchi were observed. Blood tests showed a mild elevation in white blood cells with a left shift and a slight increase in liver enzymes, but there was no significant rise in ammonia or lactate levels (Tables 1, 2). A repeat head CT scan revealed no apparent space-occupying lesions. Additionally, a chest CT scan without contrast enhancement performed to investigate hypoxemia showed intrabronchial fluid retention and ground-glass opacities in the right middle lobe, left upper lobe, and lower lobe, raising suspicion of bronchopneumonia (Figure 1). As there was no history of drug administration from symptom onset to arrival at our hospital, and the patient's level of consciousness had improved, the presentation of multiple generalized seizure episodes was considered atypical. Therefore, partial seizures, underlying schizophrenia, or drug involvement were suspected, and she was admitted for observation. Further investigations were planned, including head magnetic resonance imaging, cerebrospinal fluid analysis, and electroencephalogram.

**Table 1:** Laboratory findings. WBC: white blood cells; RBC: red blood cells; Hb: hemoglobin; Ht: hematocrit; Plt: platelets; TP: total protein; CPK: creatine phosphokinase; T-bil: total bilirubin; AST: aspartate aminotransferase; ALT: alanine aminotransferase; LDH: lactate dehydrogenase; ALP: alkaline phosphatase; AMY: amylase; NH_3_: ammonia; BUN: blood urea nitrogen; Cr: creatinine; Glu: glucose; Na: sodium; K: potassium; Cl: chloride; CRP: C-reactive protein; PT-INR: prothrombin time-international normalized ratio.

Item	Value	Reference range
WBC (/L)	12.0 × 10^9^	3.6-8.9 × 10^9^
RBC (/L)	4.07 × 10^12^	3.80-5.04 × 10^12^
Hb (g/L)	121	111-152
Ht (L/L)	0.38	0.36-0.45
Plt (/L)	221 × 10^9^	153-346 × 10^9^
TP (g/L)	68	65-85
CPK (U/L)	141	47-200
T-bil (μmol/L)	8.6	6.8-20.5
AST (U/L)	46	5-37
ALT (U/L)	38	6-43
LDH (U/L)	277	124-222
ALP (U/L)	94	38-113
AMY (U/L)	79	43-124
NH_3 _(µmol/L)	14.1	16.4-41.1
BUN (mmol/L)	4.8	3.2-7.5
Cr (µmol/L)	46.9	44.2-70.7
Glu (mmol/L)	8.54	3.61-6.05
Na (mmol/L)	142	135-145
K (mmol/L)	3.8	3.5-5.0
Cl (mmol/L)	105	96-107
CRP (nmol/L)	32.4	<28.6
PT-INR	0.96	0.9-1.1
D-dimer (μg/L)	64,000	≤1000

**Table 2:** Arterial blood gas analysis. FiO_2_: fraction of inspiratory oxygen; pCO_2_: carbon dioxide partial pressure; pO_2_: oxygen partial pressure; HCO_3_^-^: hydrogen carbonate; ABE: acid base excess; Lac: lactic acid.

Item	Value	Reference range
FiO_2 _(/L)	0.28	
pH	7.347	7.35-7.45
pCO_2_ (kPa)	5.6	4.3-6.0
pO_2 _(kPa)	6.0	11.0-14.4
HCO_3_^-^ (mmol/L)	28.8	22-26
ABE (mmol/L)	2.2	-3.3-1.2
Lac (mmol/L)	1.3	0.6-1.4

Shortly after admission, a nurse found the patient wandering within the ward and promptly assisted her back to her bed. Subsequently, the patient’s eyes rolled upward, indicating a disturbance of consciousness. The SpO_2_ was 30%, but nothing was suctioned from the oral cavity. Bag valve mask ventilation was initiated and the SpO_2_ rapidly improved to 98%, allowing the patient to nod and speak. On physical examination, she showed no signs of tachypnea and had clear breath sounds but presented with diaphoresis and mild stridor. Considering the presence of retention in the airways observed in the chest CT at admission, further aspiration was suspected, leading to the performance of a bronchoscopy. The bronchoscope was inserted through the nostrils and reached the larynx, triggering a strong cough. Beyond the vocal cords, the bronchial tree was observed, but no obvious retention was found. After the bronchoscopy, the patient was encouraged to expectorate oral contents and expulsed a red mass measuring 3 × 2 × 1.5 cm, which was identified as a watermelon eaten during breakfast on day zero.

The patient was diagnosed with aspiration pneumonia, and treatment with 2 g/day of cefotiam was initiated. By day eight, the inflammatory response had improved, and on day 10, the patient was transferred to another hospital for adjustment of psychiatric medications.

## Discussion

**Figure 2:**
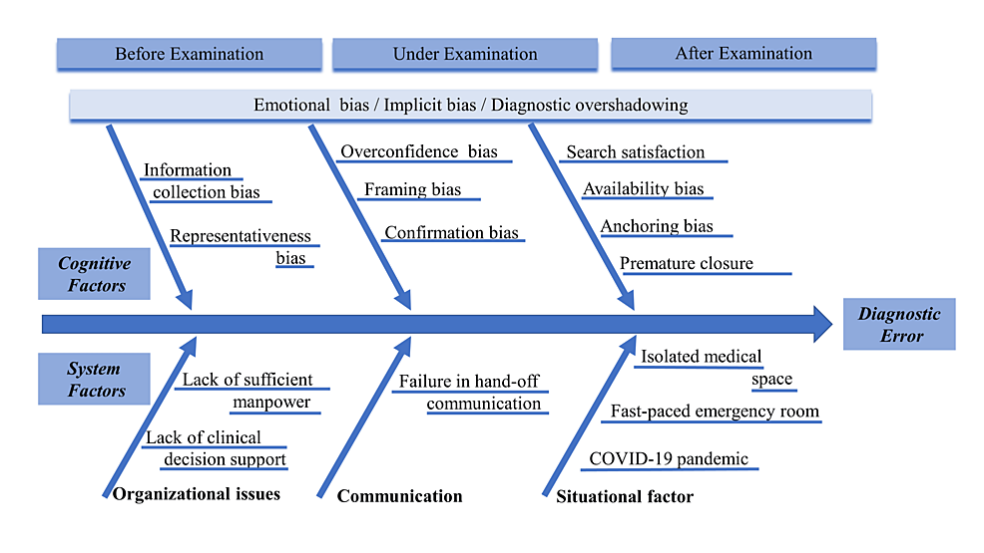
Fishbone diagram. Source: Adapted from Reilly et al. (2014) [13] under Creative Commons license (CC BY-NC-ND 3.0).

This case report describes a patient with underlying schizophrenia who experienced upper airway obstruction due to watermelon ingestion, resulting in hypoxemia, limb tremors, and ocular abnormalities that were misdiagnosed as seizure episodes, leading to two episodes of transient asphyxia. Foreign body airway obstruction is the leading cause of accidental death in Japan, and early diagnosis and removal of the causative foreign body are recommended. However, in the present case, there was a delay of approximately six hours from the onset of the patient's initial suffocation symptoms to the recognition of upper airway obstruction. Patients with schizophrenia are prone to aspiration and may have difficulty providing accurate medical histories, necessitating even more vigilance and expertise from medical professionals. When patients with psychiatric conditions experience transient hypoxemia, the possibility of upper airway obstruction should be considered during the diagnostic process. It has been reported that patients with psychiatric conditions have a significantly higher mortality rate compared with the general population, and this discrepancy is influenced not only by disparities in health care but also by diagnostic errors [10-12]. To examine the causes of diagnostic errors in this case, a fishbone diagram, commonly used in the field of patient safety, was utilized (Figure 2). This fishbone diagram was divided into cognitive and system factors, and the analysis was conducted accordingly [13].

Regarding cognitive factors, it is considered that overconfidence bias in blindly trusting the previous physician’s diagnosis and framing bias in focusing on seizure episodes and hypoxemia, as presented by the referring hospital, might have occurred. Furthermore, the influence of confirmation bias, which involves selectively gathering only favorable information, might have led to the exclusive focus on the differential diagnosis of seizure episodes without considering the possibility of hypoxemia. The satisfaction with the results of the head CT, which showed no space-occupying lesions in the cranial cavity, might have led to the denial of status epilepticus because of the lack of consciousness impairment after the seizure episodes, resulting in the exclusion of further differential diagnoses. Moreover, the experience of dealing with a psychiatric patient who was transported the previous day with suspected seizure episodes but ultimately diagnosed with drug-induced delirium could have induced availability bias in medical personnel, leading to anchoring bias and premature closure of the case designated as being another case of delirium. Additionally, the busy environment in which multiple emergency patients were treated from the medical consultation until the decision for hospitalization and the patient’s psychiatric condition might have led to emotional bias and implicit bias in healthcare providers. For patients with psychiatric conditions, diagnostic overshadowing, whereby physical symptoms are misattributed to psychiatric disorders, leading to inappropriate treatments or delayed diagnoses, is more likely to occur [14]. Factors contributing to diagnostic overshadowing include the complexity of physical expression and communication in patients with psychiatric conditions [9]. As mentioned above, anchoring, premature closure, and implicit bias can influence diagnostic overshadowing [15]. Furthermore, the lack of information about the patient’s history of ingesting watermelon, an uncommon cause of aspiration and choking, might indicate the involvement of representativeness bias in facility staff and healthcare professionals regarding information collection bias during clinical assessment. In individuals with reduced chewing ability, when consuming water-rich foods such as watermelon, the liquid part may be aspirated first, making the remaining food bolus more challenging to chew, potentially leading to choking.

Regarding the system factors in the fishbone diagram, several aspects might have contributed to the diagnostic errors in this case. First, the patient's psychiatric condition could have played a role, as individuals with schizophrenia are more prone to aspiration and have difficulty relating to a medical history. In cases where medical history cannot be obtained from the patient or their guardians, there is a higher risk of diagnostic delays, particularly when diagnosing pharyngeal foreign bodies [16]. Moreover, the busy environment of the emergency department, coupled with the emergency physician's involvement in COVID-19 treatment, might have created a physically challenging situation for seeking consultations, potentially leading to communication barriers and delays in seeking advice for this case. Additionally, chronic understaffing could have been a contributing factor. The difficulties in observing the pharynx because of the COVID-19 pandemic must have also contributed to the delay in diagnosis. Patients with schizophrenia are susceptible to dysphagia due to the effects of the disease on eating and swallowing and the impact of antipsychotic medications [7,17]. Specifically, drug-induced reduction in saliva production and decreased chewing function due to medication or the underlying condition may lead to a tendency for whole-food ingestion [18]. In the specific context of this case, it is believed that the biases depicted in the diagram influenced the decision-making process, resulting in diagnostic delays that led to two episodes of transient asphyxia and subsequent hypoxemia.

In hindsight, when status epilepticus was ruled out, there should have been a prompt suspicion of convulsions due to hypoxemia, leading to a differential diagnosis of conditions causing transient hypoxemia, such as upper airway obstruction. In the present case, the patient repeatedly performed movements to sit up, and positional changes from supine to semi-sitting were observed to improve SpO_2_ levels. When exacerbation and relief of hypoxemia were noted with changes in position, the possibility of a functional valve in the airway should be considered. While the nasal airflow was maintained in the seated position, in the supine position, the base of the tongue fell because of gravity, leading to contact between the foreign body and the posterior pharyngeal wall and consequent worsening of the airway obstruction. Despite the patient attempting positional changes to relieve upper airway obstruction, the impact of diagnostic overshadowing [14] led the medical staff to encourage the patient to remain supine, attributing the symptoms solely to the agitation associated with schizophrenia. Although the bronchoscopy failed to identify the foreign body, the rapid increase in SpO_2_ with bag valve mask ventilation at the onset of severe hypoxemia suggests that the completely obstructed airway due to the fall of the base of the tongue and the pharyngeal foreign body became unblocked owing to the pressure of airflow transmitted through coughing and the nasal passage, causing a change in the position of the foreign body. Therefore, when considering upper airway obstruction, a comprehensive examination of all upper airway regions, including the oral cavity, pharynx, larynx, trachea, and bronchi, should be conducted cautiously, and imaging studies such as CT should be performed.

## Conclusions

In conclusion, it is crucial to recognize the potential for diagnostic errors in managing patients with schizophrenia given the various biases that may come into play. Furthermore, patients with schizophrenia are at high risk of upper airway foreign body obstruction, and when transient hypoxemia is observed, a prompt assessment of the visible intraoral region and, if necessary, evaluation of the entire upper airway through imaging studies should be considered.
